# Drinking pattern is more strongly associated with under-reporting of alcohol consumption than socio-demographic factors: evidence from a mixed-methods study

**DOI:** 10.1186/1471-2458-14-1297

**Published:** 2014-12-18

**Authors:** Sadie Boniface, James Kneale, Nicola Shelton

**Affiliations:** Research Associate, HSSRG, UCL Department of Epidemiology & Public Health, 1-19 Torrington Place, London, WC1E 6BT UK; UCL Geography, London, UK; Department of Epidemiology & Public Health, London, UK

**Keywords:** Alcohol drinking, Health surveys, Public health, Mixed methods

## Abstract

**Background:**

Under-reporting of alcohol consumption is widespread; surveys typically capture 40-60% of alcohol sales. However the population distribution of under-reporting is not well understood.

**Methods:**

Mixed-methods study to identify factors associated with under-reporting, using the nationally-representative Health Survey for England (HSE) 2011 (overall response rate 66%). Comparison of retrospective computer-assisted personal interview and seven-day drinking diary (n = 3,774 adults 18+, 50% women, diary response rate 69%) to identify factors associated with diary responses exceeding those of the interview using multivariable linear regression for three outcomes: drinking days in the week recorded, volume consumed on heaviest drinking day in the week recorded, and weekly alcohol consumption. Qualitative semi-structured interviews (n = 10) explored reasons for under-reporting in further detail.

**Results:**

Number of drinking days was slightly greater in the diary than the interview (P < 0.001). Reported consumption was higher in the diary than in the interview for heaviest drinking day in the week recorded (0.7 units greater among men, 1.2 units among women, P < 0.001), and weekly alcohol consumption in women only (1.1 units among women, P = 0.003). Participants who drank more frequently, more heavily, and had a more varied drinking pattern with respect to the types of drink consumed or choice of drinking venues had a larger difference between their diary week and their interview week.

The qualitative interviews identified having a non-routine drinking pattern, self-perception as a non-frequent drinker, and usually tracking drinking using experiential approaches as linked to more drinking being reported in the diary than the retrospective interview.

**Conclusions:**

Heavy drinking and non-routine drinking patterns may be associated with greater under-reporting of alcohol consumption. Estimates of drinking above recommended levels are likely to be disproportionately under-estimated.

**Electronic supplementary material:**

The online version of this article (doi:10.1186/1471-2458-14-1297) contains supplementary material, which is available to authorized users.

## Background

Reported alcohol consumption typically amounts to 40-60% of total alcohol sales [1–6]. Comparing 2010 self-reported consumption for Great Britain (11.5 units^a^ per week per adult aged 16+ [7]) and UK alcohol sales for 2010/11 (most recent year available, 20.4 units per week per adult [8]) suggests alcohol sales coverage for the UK is 56% [9]. In 2008 the WHO’s Global Information System on Alcohol and Health estimated UK alcohol consumption (recorded + unrecorded) to be higher, equivalent to 25 UK units a week (13.24 litres per year) [10]. The discrepancy between self-reported consumption and sales is likely to be due to a combination of non-response, sampling frame issues and under-reporting.

Relatively little is known about the population distribution of under-reporting. Studies using objective or observational methods have found under-reporting was greater among participants who consumed more alcohol [11–19]. Some recent studies have used a gamma distribution to align alcohol consumption with or close to sales [20–23]. This allows the extent of under-reporting to vary by consumption level. We know of few studies that have attempted to identify wider risk factors for differences between two measures of alcohol consumption using a test-retest methodology.

The first was conducted among 49 men aged 35-45 in Finland in the early 1980s, which found the difference between participants’ quantity-frequency (QF) measure and prospective drinking diary was *not* dependent upon mean alcohol consumption in the diary, age, proportion of drinking days or the proportion of days intoxication was experienced [24]. A decade later, a study of 4,000 adults aged 20-74 in two Swedish cities used QF questions and collected information on period-specific normal week (PSNW) consumption [25]. The difference in the proportion of ‘high alcohol consumers’ between the two methods by age, education, marital status, location and occupation was greater among women than in men; with the overall proportion who were high consumers in the PSNW 182% of that in the QF for women, compared with 118% for men [25].

A 2002 Dutch study of 368 adults assessed alcohol consumption with beverage-specific QF questions and a dietary history interview [26]. Backward stepwise regression of the difference between the two measures found this was associated with a more irregular drinking pattern in beer (n = 94) and wine (n = 200) drinkers (coefficients 2.1, 95% CI 0.9-3.2 and 1.6, 95% CI 0.9-2.2 respectively), and in wine drinkers to the square of the QF response (coefficient -0.027, 95% CI -0.038 - -0.016) [26].

A 2005 Swiss study of 767 adults compared beverage-specific QF, graduated-frequency (GF) and a prospective seven-day diary [27]. Comparing the diary with the other two measures, Alcohol Use Disorders Identification Test (AUDIT) problem items were associated with higher mean consumption in the diary (difference between diary and QF = 1.00 g/day, P < 0.05, difference between diary and GF = 1.36 g/day, P < 0.01) and with inconsistencies in the alcohol consumption classification (light, low risk, hazardous, harmful) between the methods (diary compared with QF = 0.13 inconsistencies, P < 0.05, diary compared with GF = 0.19 inconsistencies, P < 0.01). In the comparison between the diary and the GF only, men reported greater mean consumption (difference of 3.13 g/day, P < 0.01) and fewer inconsistencies in classification (-0.43, P < 0.05) [27].

A 2014 study by Stockwell and colleagues made comparisons between beverage-specific ‘yesterday’ consumption and QF in the Canadian Alcohol and Drug Use Monitoring Survey [28]. They used beverage-specific ‘yesterday’ consumption to correct for under-reporting in QF and found that younger drinkers and low risk drinkers underestimated their consumption to a greater extent.

Differences between different measures arise because of poor comprehension, inaccurate recall, and deliberate misreporting of information [29]. Whilst the findings of these studies are inconsistent, they suggest demographic, social, and alcohol-related factors play a role in under-reporting. Evidence is lacking from the UK and there are no qualitative studies of this issue. This study uses data from a nationally-representative survey and in-depth interviews with survey participants to identify what role (if any) demographic, social, and alcohol-related factors have in under-reporting of alcohol consumption in England.

## Methods

### Source of data

The Health Survey for England (HSE) is an annual cross-sectional survey, representative of the adult population (aged 16+) living in private households in England. Full details of the HSE methodology are available in published reports [30]. In 2011, 66% (5,338) of sampled households took part, and 8,610 adults were interviewed [30]. As in previous years, a computer-assisted personal interview (CAPI) collected information on number of drinking days in the last week and alcohol consumption on the heaviest drinking day in the last week (recent recall).

In 2011, average weekly alcohol consumption was also calculated using beverage-specific QF questions based on the last 12 months (previously used in the General Lifestyle Survey). Adults aged 18 and over who had drunk in the last 12 months were also asked to complete a seven-day drinking diary (this began the day following the CAPI). All measures were continuous and assessed across beverage types and container sizes, where appropriate. A completed diary was returned by 69% eligible men and 71% women [31].

All analyses were weighted for non-response using the drinking diary weight for the HSE 2011. The drinking diary weight was an amended version of the main HSE interview weight that is computed by NatCen [30], which aimed to correct the distribution of the sample in terms of demographic characteristics so that the weighted sample is representative of the adults living in private households in England. The drinking diary weight amended the interview weight to allow for differential response to the diary by age, sex, household type, region, social class, smoking and general health [30]. Non-response bias remains a real possibility and the diary does not compensate for the under-representation of certain groups in the survey sample as a whole, however, there was little difference between the HSE participants and the diary subsample in terms of alcohol consumption in the CAPI.

### Quantitative sub-study

The intention was to explore differential reporting between the two measures that may be attributed to under-reporting arising from, for example, under-estimation of alcohol consumption. The sample was restricted to those who drank in both the CAPI and diary weeks (n = 3,774, 50% women). Inclusion of non-drinkers in the CAPI or diary week was considered, but they were excluded because this would bias the results in terms of making infrequent drinkers appear disproportionately inaccurate in their reporting (this group warrants detailed study in its own right). The difference between the diary and CAPI measures of drinking were calculated for three outcomes (Table 1) by subtracting the CAPI measures from those of the diary. The differences between the diary and CAPI measures were normally distributed. As the diary and the CAPI concerned two different weeks, a participant can truthfully report different consumption in the two measures. However if persistent differences between the two measures are found at a population level, risk factors for these differences warrant investigation.

**Table 1:** **Alcohol consumption measures in the HSE 2011**

Outcome	7-day drinking diary measure (prospective)	CAPI measure (retrospective)
1	Number of drinking days in the diary week (0-7)	Number of drinking days in the week prior to interview (0-7)
2	Heaviest drinking day in the diary week (units calculated from beverage specific questions with drink size)	Heaviest drinking day in the week prior to interview (units calculated from beverage specific questions with drink size)
3	Total weekly alcohol consumption (units calculated from beverage specific questions with drink size)	Average weekly alcohol consumption (units based on beverage-specific QF questions on drinking in the previous 12 months)

Multivariable linear regression investigated the extent to which demographic, social and alcohol-related variables were associated with the drinking diary exceeding the CAPI. Models presented are controlling for the other variables in the table. There were no interactions between drink type and gender for drinking frequency or weekly consumption. However for heaviest drinking day there was an interaction between gender and drink type, with women who drank wine only drinking more in their CAPI week than their diary week (coefficient -1.99, P = 0.001). There were no interactions between drinking venue and gender for drinking frequency or heaviest drinking day; however women who drank in both on and off-trade venues drank more in their CAPI week than their diary week (coefficient -3.39, P = 0.013). There were no interactions between drinking venue and age.

Likelihood ratio tests were used to assess the fit of the model with additional covariates. Covariates investigated for each of the three outcomes (but not included in final models) were: IMD quintile, equivalised household income quintile, highest educational qualification, Government Office Region, and units on heaviest drinking day in the diary week (highly correlated with total weekly units). Interactions between gender and drink type, gender and drinking venue, and age and drinking venue were also investigated. Analyses were completed in Stata 12.

### Qualitative sub-study

NatCen Social Research contacted diary participants who drank on four or more days in the diary week - for whom the issue of under-reporting may be pertinent - and who lived in one of 10 London boroughs about taking part in a subsequent research study. A maximum of one individual per household was contacted. Of 26 eligible participants contacted, one withdrew. Of the 25 whose details were passed from NatCen to the lead author, 10 were interviewed. Seven were unreachable, four declined, and four were kept as reserves. Ethical approval was obtained for the study from University College London Research Ethics Committee (reference number 2832/001). Participants were not identifiable in the HSE dataset.

Interviews primarily took place in participants’ homes (n = 9). Informed consent was obtained and a semi-structured interview schedule was used (piloted with two participants prior to use in the study), beginning with the experience of completing the drinking diary, followed by an in-depth discussion of drinking patterns and routine drinking practices. Interviews were around 45 minutes in length and were recorded. Participants received a £10 voucher as a token of appreciation. Interviews were all conducted by the lead author and took place in October and November 2012. Transcription (by the lead author) took place within two days of the interview. All interviews were completed and transcribed before they were analysed thematically using a framework approach and emic and etic coding [32]. All interviews were analysed before the quantitative analyses were conducted.

## Results

### Quantitative sub-study

#### Descriptive statistics

For both men and women, frequency and quantity of alcohol consumption were slightly higher in the diary than the CAPI. This difference was significant for all outcomes and both genders with the exception of weekly consumption among men (see Table 2).

**Table 2:** **Mean frequency or quantity of alcohol consumption in the diary and the CAPI**

	Number of drinking days*			
	**Diary**	**CAPI**		
	Mean	Mean	P for difference	*Weighted n*
**Total**	3.4	3.2	<0.001	*3,356*
**Men**	3.6	3.3	<0.001	*1,901*
**Women**	3.1	3.0	<0.001	*1,454*
	Units on heaviest drinking day*			
	**Diary**	**CAPI**		
	Mean	Mean	P for difference	*Weighted n*
**Total**	7.7	6.8	<0.001	*3,333*
**Men**	8.9	8.2	<0.001	*1,891*
**Women**	6.2	5.1	<0.001	*1,442*
	Weekly alcohol consumption**			
	**Diary**	**CAPI**		
	Mean	Mean	P for difference	*Weighted n*
**Total**	16.8	16.1	0.017	*3,796*
**Men**	20.3	19.8	0.282	*2,092*
**Women**	12.5	11.6	0.003	*1,704*

*Base = participants aged 18 and over who drank alcohol on at least one day in both the diary and CAPI weeks.

**Base = participants aged 18 and over who drank alcohol on at least one day in the diary week and reported average weekly consumption in the last 12 months. Smaller n for heaviest drinking day reflects that not all drinkers drank in CAPI week.

There were no substantive trends in the difference between the CAPI and diary measures by demographic and social factors that were consistent across the three outcomes (Additional file 1: Table S1).

The difference between the two measures by alcohol-related factors is shown in Table 3. On average, the diary exceeded the CAPI for all three outcomes among those who drank on five or more days in the diary week, those who drank more than twice the recommended limit (>8/6 units) on their heaviest drinking day in the diary, those who drank more than the weekly recommended limits (>21/14 units) in the diary, those who drank ‘other’ drinks or a combination of drink types, and those who drank in both licensed and off-licence venues in the diary week. Not surprisingly, those who said they drank ‘more than usual’ in the diary week drank more in the diary than the CAPI for all three outcomes. Most participants who completed the diary reported that the diary week was ‘about the same as usual’ (70%), with 19% reporting they drank more than usual and 11% reporting they drank less than usual.

**Table 3:** **Difference between diary and CAPI by alcohol-related variables for three outcomes in 3,774 adults aged 18 and over in the HSE 2011**

	Difference in number of drinking days (diary minus CAPI)			Difference in heaviest drinking day (diary minus CAPI)			Difference in weekly units (diary minus CAPI)		
	Men	Women	Total	Men	Women	Total	Men	Women	Total
**Drinking days (in diary week)**									
1-4 days	0.0	0.0	0.0	0.0	0.9	0.4	-0.7	0.3	-0.2
5+ days	0.8	0.9	0.8	2.3	1.8	2.1	3.6	3.0	3.4
**Units on heaviest drinking day (diary week)**									
<4/3 units	0.0	-0.1	0.0	-1.9	-1.1	-1.5	-2.8	-1.3	-2.1
>4/3 and >8/6	0.3	0.4	0.3	0.1	0.5	0.3	-1.8	-0.4	-1.1
>8/6	0.4	0.2	0.3	2.9	3.9	3.3	5.2	4.6	4.9
**Total weekly units (diary week)**									
<21/14	0.1	0.0	0.0	-0.6	0.0	-0.3	-2.4	-0.7	-1.6
>21/14	0.5	0.5	0.5	2.6	3.3	2.9	6.0	4.3	5.3
**Drink type (on heaviest day in CAPI)**									
Beer	0.3	0.3	0.3	1.7	2.4	1.8	1.7	1.9	1.7
Wine	0.3	0.2	0.2	2.3	1.3	1.6	0.0	0.3	0.2
Spirits	0.1	0.2	0.2	0.8	1.1	1.0	-2.4	0.1	-0.8
Other drinks/combination	0.3	0.3	0.3	4.3	4.2	4.3	2.8	3.1	2.9
**Drinking venue (diary week)**									
Off trade only	0.1	0.1	0.1	0.6	0.9	0.7	-1.3	0.1	-0.6
On trade only	-0.1	-0.1	-0.1	-0.1	0.5	0.1	-0.4	0.8	0.1
Mixed	0.6	0.5	0.5	1.3	1.9	1.5	4.0	2.4	3.4
**Was the diary week a usual week?**									
About the same as usual	0.1	0.1	0.1	0.4	0.5	0.4	-0.3	-0.8	-0.5
Less than usual	-0.4	-0.5	-0.5	-2.6	-0.5	-1.7	-12.9	-7.1	-10.5
More than usual	0.8	0.8	0.8	2.6	3.2	2.9	8.0	7.1	7.6

Footnote to table: Among 1,882 men (weighted = 2,147) and 1,892 women (weighted = 1,726) who drank alcohol during the diary week. Mean diff = mean difference between diary and CAPI. Positive values denote a greater diary than CAPI; negative values denote a greater CAPI than diary.

#### Multivariable analysis

Multivariable linear regression explored associations between demographic, social, and alcohol-related factors and the magnitude of the difference between the diary and the CAPI. Results are presented controlling for other variables in the table. Interactions between drink type (on heaviest day in the CAPI week) and gender, drinking venue and gender, and drinking venue and age were investigated for each outcome.

Factors associated with the number of drinking days in the diary week exceeding the CAPI (Table 4) were: the number of drinking days in the diary week (0.34 additional days, P < 0.001), drinking exclusively in the on-trade (0.17 additional days, P = 0.006), and unsurprisingly, drinking more than usual in the diary week (0.59 additional days, P < 0.001).

**Table 4:** **Factors associated with differences between the diary and the CAPI from multiple linear regression, among adults aged 18 and over in the HSE 2011**

	Difference in drinking days between diary and CAPI†				Difference in heaviest day between diary and CAPI††				Difference in weekly units between diary and CAPI†††			
	Coeff.	95% CI	P		Coeff.	95% CI	P		Coeff.	95% CI	P	
Sex (female)	-0.03	-0.15 to 0.08	0.564	ns	0.54	-0.02 to 1.09	0.057	ns	1.26	0.03 to 2.49	0.045	*
Age 25-34 (cf 16-24)	-0.07	-0.33 to 0.18	0.567	ns	-0.21	-1.78 to 1.36	0.793	ns	2.49	-1.30 to 6.28	0.197	ns
Age 35-44 (cf 16-24)	-0.26	-0.51 to 0.00	0.049	*	-0.03	-1.64 to 1.59	0.976	ns	2.01	-2.07 to 6.08	0.334	ns
Age 45-54 (cf 16-24)	-0.28	-0.53 to -0.03	0.027	*	-0.76	-2.31 to 0.79	0.338	ns	1.41	-2.81 to 5.63	0.512	ns
Age 55-64 (cf 16-24)	-0.45	-0.72 to -0.19	0.001	**	-0.04	-1.55 to 1.48	0.960	ns	1.46	-2.74 to 5.65	0.496	ns
Age 65-74 (cf 16-24)	-0.60	-0.88 to -0.31	<0.001	***	0.27	-1.27 to 1.82	0.729	ns	2.17	-2.07 to 6.41	0.316	ns
Age 75 (cf 16-24)	-0.71	-1.08 to -0.35	<0.001	***	0.34	-1.29 to 1.97	0.682	ns	2.31	-2.10 to 6.73	0.304	ns
Weekly units (diary)	-0.01	-0.02 to -0.01	<0.001	***	0.16	0.11 to 0.21	<0.001	***	0.23	0.05 to 0.42	0.015	*
Drinking days in diary week	0.34	0.29 to 0.38	<0.001	***	-0.55	-0.84 to -0.25	<0.001	***	-0.43	-1.31 to 0.46	0.344	ns
Drank wine on CAPI heaviest day (cf. beer only)	-0.10	-0.25 to 0.04	0.166	ns	0.37	-0.17 to 0.91	0.180	ns	-0.58	-2.53 to 1.38	0.562	ns
Drank spirits on CAPI heaviest day (cf. beer only)	-0.14	-0.32 to 0.05	0.155	ns	0.18	-0.62 to 0.99	0.652	ns	-0.69	-2.73 to 1.36	0.510	ns
Drank other drinks/combination on CAPI heaviest day (cf. beer only)	-0.30	-0.51 to -0.09	0.006	**	1.23	0.36 to 2.10	0.006	**	0.94	-0.86 to 2.75	0.305	ns
Drank in on trade only in diary week (cf. off trade only)	0.17	0.02 to 0.32	0.024	*	-0.18	-0.84 to 0.49	0.605	ns	1.85	0.53 to 3.17	0.006	**
Drank in on & off trade in diary week (cf. off trade only)	0.11	-0.03 to 0.26	0.122	ns	0.44	-0.10 to 0.98	0.108	ns	1.93	0.34 to 3.52	0.018	*
Drank less than usual in diary week (cf. same as usual)	-0.36	-0.61 to -0.12	0.004	**	-2.18	-3.09 to -1.27	<0.001	***	-10.08	-14.59 to -5.58	<0.001	***
Drank more than usual in diary week (cf. same as usual)	0.59	0.45 to 0.73	<0.001	***	1.62	0.97 to 2.27	<0.001	***	6.09	4.66 to 7.53	<0.001	***

Footnote to table: Coefficients from multiple linear regression accounting for complex survey design and using the drinking diary weight. Coeff. = coefficient, HDD = heaviest drinking day, P = p-value, ns = not significant. * = P < 0.05, ** = P < 0.01, *** = P < 0.001. † = among 2,722 adults with full information on all covariates. †† = among 2,700 adults with full information on all covariates. ††† = among 3,135 adults with full information on all covariates.

Factors associated with the diary heaviest drinking day exceeding the CAPI were: weekly alcohol consumption in the diary week (for every extra unit consumed in the diary week, the diary exceeded the CAPI by 0.16 additional units, P < 0.001), drinking a combination of drink types (1.23 additional units, P = 0.006), and having drunk more than usual in the diary week (1.62 additional units, P < 0.001).

Factors associated with weekly alcohol consumption in the diary exceeding the CAPI were: weekly alcohol consumption in the diary (for every extra unit consumed in the diary week, the diary exceeded the CAPI by 0.23 additional units, P = 0.015), drinking exclusively in the on-trade (1.85 additional units, P = 0.006) and drinking in both the on and off trade (1.93 additional units, P = 0.018), and, again, drinking more than usual in the diary week (6.09 additional units, P < 0.001). Gender was borderline significant (P = 0.045), with women reporting on average 1.26 extra units weekly in the diary compared with what was reported in the CAPI.

#### Qualitative sub-study

The 10 participants (7 men, 3 women) were aged 25-90 years. Nine were employed at the time of the HSE interview. Participants lived in areas of varying deprivation (IMD quintile), although most were from the highest two income quintiles (N.B. two had withheld this information). Most participants said the week they completed the diary was, or probably was, a fairly typical week. All stated that the drinking diary did not influence their alcohol consumption during that week. Some had changed their drinking patterns somewhat since, all said this was unconnected to the diary.

#### Mode effects: social desirability and recall

Effects of survey mode were apparent; participants made a clear distinction between the diary and the CAPI questions about drinking which reflect a social desirability bias in speaking to an interviewer about alcohol. Six participants said that the anonymity of the self-completion diary led to increased honesty. These participants believed they were likely to make generalisations in the CAPI, compared with the diary:

*If somebody asks you, like about your drinking habits I think you automatically put up a sort of, a little bit defensive and a little bit glossy on um, what you do…I suppose it’s more truthful* [the diary]*, it’s more, like because it’s there in black and white…it would just be, as it is I s’pose. Rather than, um, finessing certain bits or missing certain bits out.*

Male, age 35-44, most deprived area quintile, second highest income quintile, employed.

Another issue of the survey mode was recall. As the diary was completed in closer proximity to the drinking occasion, recall was easier:

*You do it, sort of, much quicker after the event. Much more proximity to the event. I mean even if you’d sat down and at the end of the week tried to remember you’d have struggled.*

Female, age 45-54, second most deprived area quintile, highest income quintile, employed.

Although there was a consensus across the interviews that the diary was a more objective - a ‘better’ measure of drinking - the reasons behind this varied, suggesting that the reasons for under-reporting are complex.

### Actual and perceived drinking pattern

Half of participants described their drinking pattern as routine. These participants generally felt they had a good idea of how much they drank before they did the diary:

*I knew exactly, pretty well, what I was drinking and I was aware for some time that I was probably exceeding, certainly exceeding 21 units…no I’m quite open about it.*

Male, 75+, least deprived area quintile, second highest income quintile, retired.

Routine drinkers were often older, and did most of their drinking at home. Participants with a varied drinking pattern more commonly recalled experiencing some element of surprise at their alcohol consumption when they completed the diary. Although this surprise concerned the quantity of alcohol consumed to an extent, frequency of drinking was of particular mention, and this often conflicted with participants’ perceptions:

*It was actually the fact that sort of done* [sic] *two or three days in the week where I also drank. Whereas normally I think ‘I don’t drink for four days a week so it’s fine’.*

Male, 25-34, middle deprivation area quintile, highest income quintile, employed.

*I tend to think of myself as somebody who just drinks, you know, maybe Friday and Saturday, and sometimes I thought ‘ooh, I’ve actually had four nights when I’ve had some alcohol this week’.*

Female, age 45-54, second most deprived area quintile, highest income quintile, employed.

Having a non-routine drinking pattern and inaccurate perceptions of that pattern were both linked to drinking more in the diary than expected, and could contribute to under-reporting.

### Usual methods of tracking drinking

Usual methods by which participants tracked their drinking were also associated with whether they experienced any surprise at their alcohol consumption when they completed their diary. Only one participant said units were a helpful way of tracking drinking. Most commonly drinking was tracked as numbers of drinks or fractions of bottles. Put simply:

*If the bottle’s empty I know I drank a bottle, don’t I?!*

Male, 65-74, least deprived area quintile, income withheld, employed.

Counting drinks was frequently combined with other methods. Of the participants who exclusively used units or counted drinks (n = 5) to track their drinking, few said that they recorded higher alcohol consumption than they would have expected in their drinking diary. Participants who used more experiential approaches to track their consumption tended to recall drinking either larger quantities of alcohol or more frequently than they expected when they completed their drinking diary. Experiential approaches included embodied aspects (perceived level of intoxication) or individualised approaches. Participants who used these approaches to track drinking had varying drinking patterns. Embodied approaches are exemplified by participants who sought certain pleasurable levels of intoxication, with the amount of alcohol consumed to reach that point of little relevance:

*Friday and Saturday probably not [able to estimate drinks]. You know I wouldn’t be able to put a number on it…it’s how you feel on it. You know, it’s, you get that nice little buzz.*

Male, aged 45-54, second least deprived area quintile, highest income quintile, employed.

Individualised approaches were used by two men interviewed. For them, these were a tool used in their daily lives. One participant divided drinking occasions into lighter and heavier using a three-pint rule. For him, drinking was tracked by counting drinks if he had three pints of beer or less, but when drinking more than three pints consumption was no longer relevant (it was just ‘a big night’). The other participant’s approach used was a continuum, whereby consumption was estimated based on the time elapsed since the start of the drinking occasion. The two participants using individualised approaches to track their drinking were quite aware that these had limitations, and the origin of these approaches was unclear. These findings regarding experiential approaches to tracking drinking suggest people who track their drinking in these ways may be more prone to under-report their alcohol consumption.

## Discussion

The differences between the prospective and retrospective measures of drinking in the HSE 2011 were modest. Even so, some alcohol-related factors were associated with the diary estimates exceeding those of the CAPI; these included consumption level as well as drink type and drinking venue. In the qualitative interviews, participants with non-routine drinking patterns or using experiential approaches to track drinking found the diary particularly revealing. Together, these findings suggest that alcohol-related factors require further investigation if the population distribution of under-reporting is to be better understood. The findings of this study are not at all consistent with those of recent Canadian study by Stockwell and colleagues [28]. Although the methods used in Stockwell’s study and the present study are quite different (for example our study was not designed to detect differences among people who do not drink every week), it would have been anticipated that the findings would have been at least to some extent similar.

### Strengths

Previous studies which have identified factors associated with a difference between two measures of drinking were small (<1,000 participants) [24, 26, 27], or did not attempt to identify risk factors beyond basic demographic factors [25]. Alcohol-related factors were previously identified as important [26, 27]. This study corroborates these earlier findings, but is the first such study to be conducted in England. As the HSE was a nationally-representative sample, these findings are broadly generalisable across England.

The qualitative component is a new approach to a quantitative and methodological problem; the findings suggest that a mixed methods approach is potentially useful in this area, and that qualitative research has a place in understanding under-reporting. The fact that participants could volunteer their experiences and ideas about doing the diary has led to a richer and more nuanced understanding of *why* alcohol consumption is under-reported in social surveys.

### Limitations

Response to the diary was 69%, and although CAPI measures of alcohol consumption were similar in the diary subsample and the full HSE sample, it is possible that non-responders to the diary were systematically different to responders. A drinking diary weight was computed to adjust for non-response, however, it has been suggested that it may be preferential to use the continuum of resistance model which takes into account the amount of time and effort required to elicit a response (after Lin and Schaeffer [33]) to adjust for non-response than population weights [34].

It was obviously not possible to identify risk factors for under-reporting the alcohol consumption that was not captured by the diary. A large difference between consumption reported in the diary and alcohol sales remains; if the diary estimates were used alcohol sales coverage would not improve much. The risk factors identified do not explain why alcohol sales coverage is low but they do provide clues for the direction of future research.

The quantitative analyses make intra-individual comparisons between two measures of drinking. However, this not comparing drinking in the same week: the diary week and the CAPI week were two different weeks. Therefore our intra-individual comparison is of little value on an individual level. Collectively, however, it is possible to observe whether there is a tendency for diary measures of drinking to exceed those of the CAPI. We restricted our analyses to those who drank in both the diary and the CAPI therefore our findings may be biased by the exclusion of infrequent drinkers.

A small number of qualitative interviews was conducted due to resource constraints in terms of the cost of selecting HSE participants through NatCen as well as travel and time. A random sample of 10 individuals would have been cheaper and quicker, but we wanted to explore HSE participants’ unique experience of having completed the drinking diary and CAPI. Saturation was not reached, and it is possible that more - or conflicting - factors would emerge in further interviews. Participants were mainly men (70%) and relatively affluent (which may be somewhat driven by selection on drinking on four or more days). However it is not the aim of qualitative research to produce findings that are generalisable – the intention was to identify factors that it was not possible to investigate in the quantitative data. The ten interviews conducted were a rich source of data and both supplement and complement the quantitative study.

The qualitative interviews were carried out in late 2012, 11-20 months after the original HSE 2011 CAPI and drinking diary took place, so responses may be affected by poor recall. Participants were however recruited as soon as it was possible to select participants from the HSE 2011 dataset. Future studies might recruit participants from the general population to complete a diary and then conduct interviews with these participants in closer proximity to consumption taking place.

## Conclusion

Alcohol-related factors linked to greater quantities of alcohol consumption and a more varied drinking pattern were associated (to varying extents) with under-reporting of alcohol consumption as interpreted as a difference between the CAPI and diary measures of drinking. It is concerning that the burden of under-reporting may fall on heavier drinkers. Estimates of the prevalence of drinking above the recommended levels, hazardous levels, or harmful levels based on self-reported consumption may be disproportionately under-estimated.

There is evidence that participants who are late responders to surveys have more risky health behaviours (e.g. binge drinking and non-compliance with physical activity levels) than those who respond earlier [35, 36]. Although non-response bias was not explored in detail in this study, it would be valuable to explore this using the continuum of resistance model in a UK sample in future research. The absence of demographic and social factors that were associated with for under-reporting suggests specific research may be necessary to understand the effects of non-response and under-reporting on other self-reported health behaviours as well, including: sexual behaviours, drug taking, tobacco smoking, diet and physical activity.

## Endnote

^a^One UK unit = 10 ml or 8 g pure alcohol (EtOH)

## Electronic supplementary material

Additional file 1: Table S1: Difference between diary and CAPI by sociodemographic factors for three outcomes in 3,774 adults aged 18 and over in the HSE 2011. (DOCX 23 KB)
