# Supplementary material for: Drinking pattern is more strongly associated with under-reporting of alcohol consumption than socio-demographic factors: evidence from a mixed-methods study
Source: BMC Public Health. 2014 Dec 18;14:1297. doi: 10.1186/1471-2458-14-1297 (PMC4320509; doi:10.1186/1471-2458-14-1297)
Supplement: Supplementary file 1 — Additional file 1: Table S1: Difference between diary and CAPI by sociodemographic factors for three outcomes in 3,774 adults aged 18 and over in the HSE 2011. (DOCX 23 KB) [file 12889_2014_7460_MOESM1_ESM.docx]

Additional file Table S1

|  | **Difference in number of drinking days (diary-CAPI)** | | | **Difference in heaviest drinking day (diary-CAPI)** | | | **Difference in weekly units (diary-CAPI)** | | |
| --- | --- | --- | --- | --- | --- | --- | --- | --- | --- |
|  | **Men** | **Women** | **Total** | **Men** | **Women** | **Total** | **Men** | **Women** | **Total** |
| Age (10 year bands) | | | | | | | | | |
| 16-24 | 0.3 | -0.2 | 0.1 | -1.3 | 1.6 | 0.1 | -0.5 | -0.6 | -0.6 |
| 25-34 | 0.3 | 0.3 | 0.3 | 0.1 | 0.3 | 0.1 | 0.6 | 1.8 | 1.1 |
| 35-44 | 0.2 | 0.3 | 0.3 | 0.8 | 1.5 | 1.1 | 0.6 | 2.3 | 1.4 |
| 45-54 | 0.4 | 0.2 | 0.3 | 0.5 | 1.3 | 0.9 | 1.2 | 0.2 | 0.7 |
| 55-64 | 0.2 | 0.2 | 0.2 | 1.7 | 1.3 | 1.5 | 0.6 | 0.8 | 0.7 |
| 65-74 | 0.3 | -0.1 | 0.1 | 1.5 | 0.9 | 1.3 | 0.8 | 0.0 | 0.5 |
| 75+ | 0.1 | 0.1 | 0.1 | 1.4 | 0.5 | 1.0 | 0.0 | -0.8 | -0.4 |
| Equivalised household income quintile | | | | | | | | | |
| 1 (lowest) | 0.4 | 0.1 | 0.3 | 0.0 | 1.5 | 0.7 | 1.1 | -0.2 | 0.5 |
| 2 | 0.3 | 0.2 | 0.2 | 0.5 | 0.5 | 0.5 | -0.3 | 0.3 | 0.0 |
| 3 | 0.1 | 0.3 | 0.2 | 1.2 | 1.1 | 1.1 | 0.4 | 1.4 | 0.9 |
| 4 | 0.1 | 0.2 | 0.1 | 1.0 | 1.6 | 1.3 | 0.8 | 0.9 | 0.9 |
| 5 (highest) | 0.3 | 0.2 | 0.3 | 0.3 | 0.9 | 0.6 | 2.0 | 1.4 | 1.7 |
| IMD quintile | | | | | | | | | |
| 1 (least deprived) | 0.2 | 0.2 | 0.2 | 1.0 | 0.9 | 1.0 | 0.3 | 0.9 | 0.6 |
| 2 | 0.3 | 0.1 | 0.2 | 1.0 | 1.0 | 1.0 | 0.4 | 0.4 | 0.4 |
| 3 | 0.2 | 0.2 | 0.2 | 0.4 | 1.1 | 0.7 | 0.5 | 1.0 | 0.7 |
| 4 | 0.2 | 0.2 | 0.2 | 0.8 | 0.9 | 0.8 | 1.8 | 1.4 | 1.6 |
| 5 (most deprived) | 0.3 | 0.1 | 0.2 | -0.1 | 2.0 | 0.7 | -0.1 | 0.3 | 0.1 |
| Region | | | | | | | | | |
| North East | 0.2 | 0.1 | 0.2 | 0.9 | 0.6 | 0.8 | 1.9 | 0.9 | 1.5 |
| North West | 0.3 | 0.2 | 0.3 | -0.2 | 0.9 | 0.3 | 0.9 | -0.5 | 0.3 |
| Yorkshire and The Humber | 0.2 | 0.1 | 0.2 | 1.4 | 0.8 | 1.1 | 2.1 | 1.1 | 1.6 |
| East Midlands | 0.4 | 0.0 | 0.2 | 1.2 | 1.5 | 1.3 | -3.3 | -0.3 | -1.8 |
| West Midlands | 0.3 | 0.2 | 0.2 | -0.3 | 0.8 | 0.2 | 0.0 | -1.7 | -0.7 |
| East of England | 0.4 | 0.2 | 0.3 | 0.7 | 1.2 | 1.0 | 1.6 | 2.3 | 1.9 |
| London | 0.2 | 0.4 | 0.3 | 0.0 | 1.1 | 0.5 | 0.1 | 2.4 | 1.0 |
| South East | 0.2 | 0.1 | 0.1 | 1.1 | 1.1 | 1.1 | 0.2 | 1.4 | 0.7 |
| South West | 0.1 | 0.3 | 0.2 | 1.8 | 1.7 | 1.8 | 1.8 | 1.2 | 1.5 |
| Highest educational qualification | | | | | | | | | |
| NVQ4/NVQ5/Degree equiv | 0.4 | 0.2 | 0.3 | 0.2 | 1.2 | 0.7 | -0.2 | 1.7 | 0.7 |
| Higher education below degree | 0.1 | 0.0 | 0.1 | 0.5 | 1.1 | 0.7 | 0.7 | -0.5 | 0.2 |
| NVQ3/GCE A Level equiv | 0.3 | 0.4 | 0.3 | 0.8 | 1.0 | 0.9 | 1.4 | 0.7 | 1.1 |
| NVQ2/GCE O Level equiv | 0.2 | 0.3 | 0.2 | 1.6 | 1.7 | 1.6 | 1.2 | 1.0 | 1.1 |
| NVQ1/CSE other grade equiv | -0.1 | 0.2 | 0.0 | -1.0 | 0.1 | -0.6 | 1.9 | 0.4 | 1.4 |
| Foreign/other | 1.0 | -0.5 | -0.5 | -0.2 | -0.7 | -0.7 | -3.8 | -1.2 | -1.4 |
| No qualification | 0.2 | 0.2 | 0.2 | 1.1 | 0.9 | 1.0 | -1.1 | 0.4 | -0.4 |
| Full-time student | 0.4 | -0.1 | 0.2 | 0.5 | 0.4 | 0.4 | 3.5 | 0.4 | 2.1 |

*Footnote to table: Among 1,882 men (weighted = 2,147) and 1,892 women (weighted = 1,726) who drank alcohol during the diary week and the CAPI week. Mean diff = mean difference between diary and CAPI. Positive values denote a greater diary than CAPI, negative values denote a greater CAPI than diary.*
